# Catalytic Effect of Pd Clusters in the Poly(*N*-vinyl-2-pyrrolidone) Combustion

**DOI:** 10.1186/s11671-017-2422-0

**Published:** 2018-01-11

**Authors:** L. Schiavo, S. De Nicola, G. Carotenuto

**Affiliations:** 1 Physics Department, University of Trento, Via Sommarive, 14, 38123 Povo, Italy; 2 Institute for Polymers, Composites and Biomaterials (IPCB), National Research Council (CNR), Piazzale E. Fermi, 1, 80055 Portici, NA Italy; 3 CNR-SPIN, Institute for Superconductors, Innovative Materials and Devices, (CNR-SPIN) Napoli, Complesso Universitario di M.S. Angelo, Via Cinthia, 80126 Naples, Italy

**Keywords:** Heterogeneous catalysis, Palladium, Combustion, Poly(*N*-vinyl-2-pyrrolidone), Thermogravimetry

## Abstract

Pd(0) is able to catalyze oxygen-involving reactions because of its capability to convert molecular oxygen to the very reactive atomic form. Consequently, the embedding of a little amount of Pd(0) clusters in polymeric phases can be technologically exploited to enhance the incineration kinetic of these polymers. The effect of nanostructuration on the Pd(0) catalytic activity in the polymer incineration reaction has been studied using poly(*N*-vinyl-2-pyrrolidone) ($\overline{Mw}$ = 10,000 gmol^−1^) as polymeric model system. A change in the PVP incineration kinetic mechanism with significant increase in the reaction rate was experimentally found. The kinetic of the Pd(0)-catalyzed combustion has been studied by isothermal thermogravimetric analysis. After a short induction time, the combustion in presence of Pd(0) clusters shifted to a zero-order kinetic from a second-order kinetic control, which is operative in pure PVP combustion reaction. In addition, the activation energy resulted much lowered compared to the pure PVP incineration case (from 300 to 260 kJ/mol).

## Background

On a nanoscopic scale, noble metals show abnormally increased catalytic properties, known as “super-catalytic effect” [1, 2]. Not only the number of catalytic sites increases with the decreasing of the crystal diameter, due to the surface/volume ratio increase (the surface/volume ratio for a spherical particle is 3/R), but also the catalytic site nature (that is, Lewis’ acidity) is strongly affected [1]. In particular, Lewis’ acidity of catalytic sites increases with size decreasing because the relative abundance of different catalytic sites changes. According to theoretical models, like for example the “cubic model” [3], the distribution of the different site types (i.e., basal plane, edge, and corner sites) in a noble metal crystal is revolutionized by decreasing the crystal diameter [1]. In fact, in some micron powders, crystal basal planes are the most abundant, while edge and/or corner sites prevail in a nanoscopic crystal system [2]. Owing to the lower coordination number of these sites, a different catalytic activity follows. Moreover, as the activity also, selectivity and specificity of catalytic sites are modified [4, 5].

Polymer incineration is a technologically relevant chemical process, which involves oxygen and takes place at relatively high temperatures [6, 7]. PVP is a common polymer, industrially exploited in several fields (cosmetics, biomedical, as excipient in drugs, etc.), and hence, it was selected as the “model polymer” to be studied. In addition, the PVP incineration is technologically relevant in ceramic sintering [8], ceramic sensor fabrication [8, 9], battery electrode fabrication [6], waste destruction [10, 11], solid propellant development [12, 13], etc.

Here, we have found that the PVP incineration process can benefit of the presence of a nano-sized noble metal catalyst, probably because it is able to quantitatively convert molecular oxygen (O_2_) to the more aggressive oxygen atoms (O·) [14, 15]. All types of noble metal/PVP combinations can be easily synthesized, in a high homogeneous form, by using the very common polyol process technique [16–22]. In this study, Pd has been selected as a catalytic metal since it can be achieved in an extremely small size by this simple reaction scheme [21, 22].

Polymer incineration can be readily studied by using thermogravimetric analysis (TGA) [7]. In particular, isothermal TGA tests, performed at temperatures higher than the PVP ignition temperature, have been used for this kinetic analysis. The isothermal TGA tests were performed at four different temperatures slightly above 370 °C, which corresponds to the onset degradation temperature in a dynamic TGA thermogram. Temperatures higher than 440 °C were not investigated because the reaction rate resulted too high for a satisfactory TGA monitoring. In order to establish the involved combustion mechanism, (i) the reaction order, (ii) kinetic constant, (iii) and activation energy values have been valuated from the isothermal TGA data of pure PVP and nano-Pd/PVP combustions.

## Experimental

Samples were prepared according to a literature method [22]. In particular, poly(*N*-vinyl-2-pyrrolidone) (PVP, Aldrich, $\overline{Mw}$ = 10,000 gmol^−1^) was dissolved in dry ethylene glycol (EG, Aldrich, 99.8%), and the solution was placed in a thermostatic bath at 90 °C in air, up to complete dissolution. In a typical preparation, 24 g of PVP powder were dissolved into 70 ml of EG. Separately, a smaller volume (10 ml) of a concentrated solution of potassium tetrachloropalladate(II) (K_2_PdCl_4_, Aldrich, 99.99%) in EG (0.35% by weight of salt) was prepared, and it was rapidly injected into the vigorously stirred hot PVP/EG solution. The PVP concentration in EG was 30 mM and the Pd(II):PVP molar ratio was 1:10. After heating for 120 min, the solution was cast into a large amount of acetone to flocculate the nano-Pd/PVP system. The product was dried in air and stored at room temperature in a desiccator. A second type of nano-Pd/PVP samples was also prepared by dispersing a commercial micrometer-sized Pd(0) powder (Pd, Aldrich, particles size < 1 μm, 99.9%) in PVP having the same molecular weight. The dispersion concentration was similar to that used for the nano-sized Pd in PVP samples (0.3% by weight).

The morphology of Pd clusters was investigated in a PVP-embedded form by using transmission electron microscopy (TEM, FEI Tecnai G2 Spirit twin apparatus) operated at 120 kV, and after the combustion process, the residual product was imaged by using scanning electron microscopy (SEM, FEI QUANTA 200 FEG apparatus).

According to the literature [23, 24], the combustion properties of pure poly(*N*-vinyl-2-pyrrolidone) (PVP) and nano-Pd/PVP samples were investigated by thermogravimetric analysis (TGA, Q5000, TA Instruments) in oxidative atmosphere (fluxing air) at standard pressure conditions. The combustion process was investigated by burning PVP and nano-Pd/PVP samples in both dynamic (from room temperature to 600 °C, at a heating rate of 10 °C min^−1^) and isothermal conditions, under fluxing air (25 mL min^−1^). The temperature of TGA isothermal tests was taken above the ignition temperature (onset) determined by the TGA dynamic scan. Isothermal data were recorded for all samples up to a complete weight loss.

## Results and Discussion

**Fig. 1:**
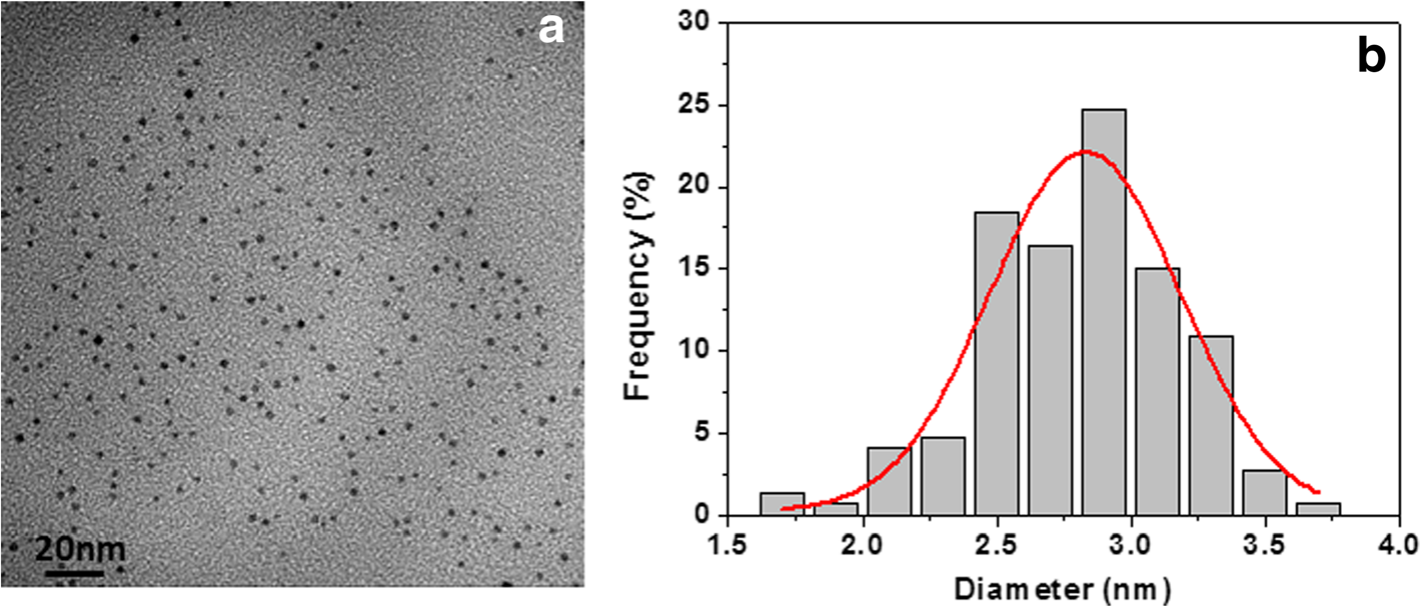
TEM micrograph of nano-Pd/PVP sample (**a**) and particle size distribution (**b**)

A representative TEM micrograph of nano-Pd/PVP samples is shown in Fig. 1a. Contact-free Pd clusters, having a size of 2.8 ± 0.2 nm (see Fig. 1b), appear to be uniformly dispersed into the PVP matrix.

**Fig. 2:**
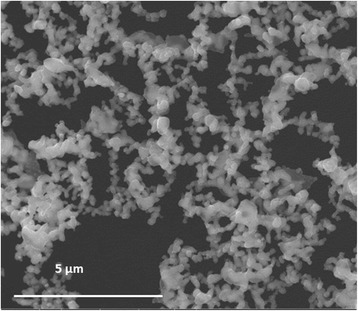
SEM micrograph of the residual TGA product (palladium powder)

The SEM micrograph shown in Fig. 2 evidences as, in presence of the Pd(0) catalyst, the PVP combustion resulted complete. In fact, the combustion product consisted of only aggregated Pd clusters, without traces of any organic residue. In particular, this nano-Pd/PVP sample was burned in a thermogravimetric balance, under dynamic conditions (i.e., from room temperature to 600 °C, at a heating rate of 10 °C min^−1^), using fluxing air (25 ml min^−1^). The continuous metallic framework has been generated by the sintering of adjacent Pd(0) clusters after the PVP removal.

**Fig. 3:**
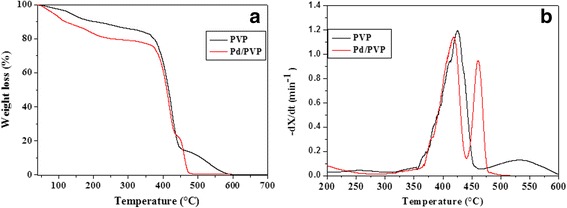
Weight loss (**a**) and weight loss rate (**b**) corresponding to the combustion of pure PVP (black) and nano-Pd/PVP (red), performed at a heating rate of 10 °C min^−1^

The combustion processes for pure PVP and nano-Pd/PVP samples were studied by dynamic and isothermal thermogravimetric analysis (TGA). Dynamic TGA allowed to determine at which temperature the combustion starts (i.e., the ignition temperature) and ends, and it provided general information regarding reaction kinetics and other parameters that characterize the combustion behavior. A comparative analysis of the TGA (weight loss ratio) and DTG (weight loss rate) curves is presented in Fig. 3 for pure PVP and nano-Pd/PVP samples. The shape of the curves indicates that the major weight loss occurs between 400 and 500 °C. Pure PVP and nano-Pd/PVP samples differ in reactivity as clearly exhibited by the deviations in peak decomposition rate and by the trend of the weight loss curve of nano-Pd/PVP which on the whole is faster compared to that of pure PVP. In fact, the presence of a very small amount of Pd catalyst affects the polymer thermal degradation both before and after ignition (see Fig. 3a). Further, a residual weight loss of ca. 0.3%, due to the Pd catalyst content, is clearly visible in the nano-Pd/PVP TGA. The DTG curves of pure PVP and nano-Pd/PVP samples exhibit a maximum decomposition rate at ca. 420 °C (see Fig. 3b). In addition, the nano-Pd/PVP curve displays a second peak which is visibly anticipated compared to the pure PVP (470 °C instead of 540 °C).

**Fig. 4:**
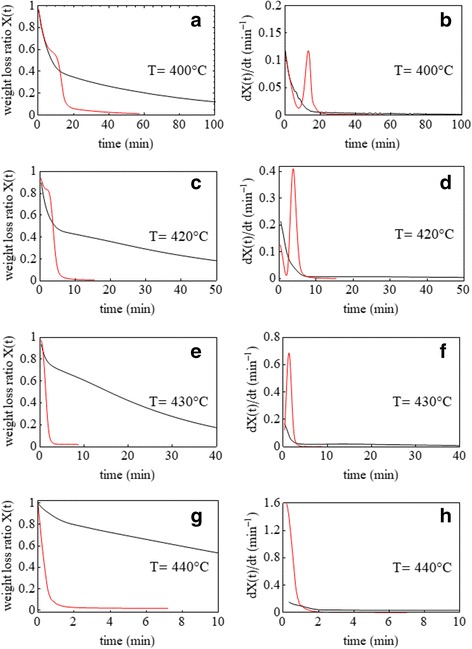
Isothermal weight loss ratio as a function of time during combustion of pure PVP (black curves) and nano-Pd/PVP (red curves) at 400 °C (**a**), 420 °C (**c**), 430 °C (**e**), and 440 °C (**g**) and corresponding derivative curves (**b**, **d**, **f**, **h**)

TGA isothermal analysis was used to study the combustion kinetic of the PVP catalyzed by Pd(0). Figure 4 shows the isothermal weight loss fraction, *X*(*t*), as a function of time during the combustion process for pure PVP and nano-Pd/PVP samples at temperatures slightly above the ignition temperature. The weight loss fraction is defined as *X*(*t*) = *w*(*t*)/*w*_0_, where *w*_0_ and *w*(*t*) refer to the initial weight and weight at time *t*, respectively. Isothermal thermogravimetric analysis was carried out at four different temperatures: 400, 420, 430, and 440 °C.

Two combustion stages are visible in the thermograms (see Fig. 4): in the first stage, a similar weight loss behavior, characterized by perfect curves that overlap, is found for both pure PVP and nano-Pd/PVP samples. This stage tends to become shorter with temperature increasing. In the second stage, the nano-Pd/PVP weight loss curve decreases very rapidly and then level off to become asymptotic to the residual Pd content. These two stages are linked together through a short time interval in which the weight loss keeps almost constant.

Such behavior can probably be ascribed to the time needed to saturate the Pd surface by molecular oxygen [9]. Pd catalyst is able to quantitatively convert the low-reactive molecular oxygen (O_2_) to a very reactive atomic oxygen species. Therefore, in the first stage, common to both PVP and nano-Pd/PVP combustion, only the molecular oxygen plays the dominant role in the reaction while atomic oxygen plays a major role in the second stage of nano-Pd/PVP combustion.

The weight loss curve of pure PVP reaches its asymptotic value only after a very long time, and according to the applied regression analysis, it follows a second-order kinetic behavior. Differently, the nano-Pd/PVP curves quickly drop to their asymptotic values (see Fig. 4) following a linear behavior, thus suggesting a zero-order kinetic control for this second stage of combustion. In particular, a correlation factor of *R*^2^ = 0.98 was found for all curves. The estimated values of kinetic constants both in presence and absence of the Pd(0) catalyst are given in Table 1 for each combustion temperature.

**Table 1:** Kinetic constants of pure PVP and nano-Pd/PVP combustion at different temperatures

Temperature (°C)	*k* (min^−1^) for nano-Pd/PVP combustion	*k* (min^−1^) for pure PVP combustion
400	0.113	0.041
420	0.409	0.152
430	0.711	0.324
440	1.632	0.886

**Fig. 5:**
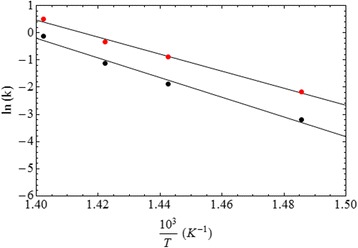
Arrhenius plot for pure PVP (black points) and nano-Pd/PVP samples (red points)

In order to determine the activation energy for the fast stage of the nano-Pd/PVP combustion process, the *k = A·exp(−E*_*a*_*/RT)* Arrhenius equation has been used to fit the kinetic constants at different temperatures. The constant *A* is the frequency factor, *E*_a_ is the activation energy, and *R* is the gas constant. The Arrhenius plot (ln(k) vs. 1/T) is given in Fig. 5. The solid line is the linear fit of the experimental kinetic constants, and its slope depends on the activation energy (*E*_a_). The calculated pre-exponential factor, *A*, was 1.7 × 10^19^ min^−1^ (ln(A) = 44.3), and the activation energy (*E*_a_) was ca. 260 kJ/mol. For comparison, Fig. 5 shows also the Arrhenius plot of the kinetic constants for pure PVP combustion. The calculated pre-exponential factor *A* was 7.7 × 10^21^ min^−1^ (ln(A) = 50.4), and the activation energy (*E*_a_) was ca. 300 kJ/mol, determined from the slope of the linear fit to the kinetic constants (black dots) of the PVP combustion.

According to Fig. 5 and to the data shown in Table 1, the presence of the palladium catalyst has the global effect of lowering the activation energy of the polymer combustion, allowing the reaction to speed up (higher kinetic constant values).

The knowledge of the activation energy, *E*_a_, helps to develop hypothesis on the reaction mechanism. In fact, by comparing *E*_a_ with the tabulated values of bond energies, one can determine which is the rate-limiting step in the combustion reaction under investigation. The calculated activation energy, *E*_a_ = 260 kJ/mol, is about one half the molecular oxygen double bond energy (498.36 ± 0.17 kJ/mol) [25]. Since the single-bond energy of oxygen is quite close to the measured activation energy, it can be concluded that atomic oxygen formation *(O*_*2*_ *→ 2O)* takes place on the palladium cluster surface and it is the rate-limiting step of the nano-Pd/PVP combustion. In fact, under fluxing oxygen condition (constant oxygen concentration), the kinetic order of this elementary process is just like zero. Other elementary processes involved in the catalyzed combustion mechanism, which can be schematically indicated as *PVP + O → gaseous products*, should take place on a time scale much faster than the atomic oxygen formation step.

**Fig. 6:**
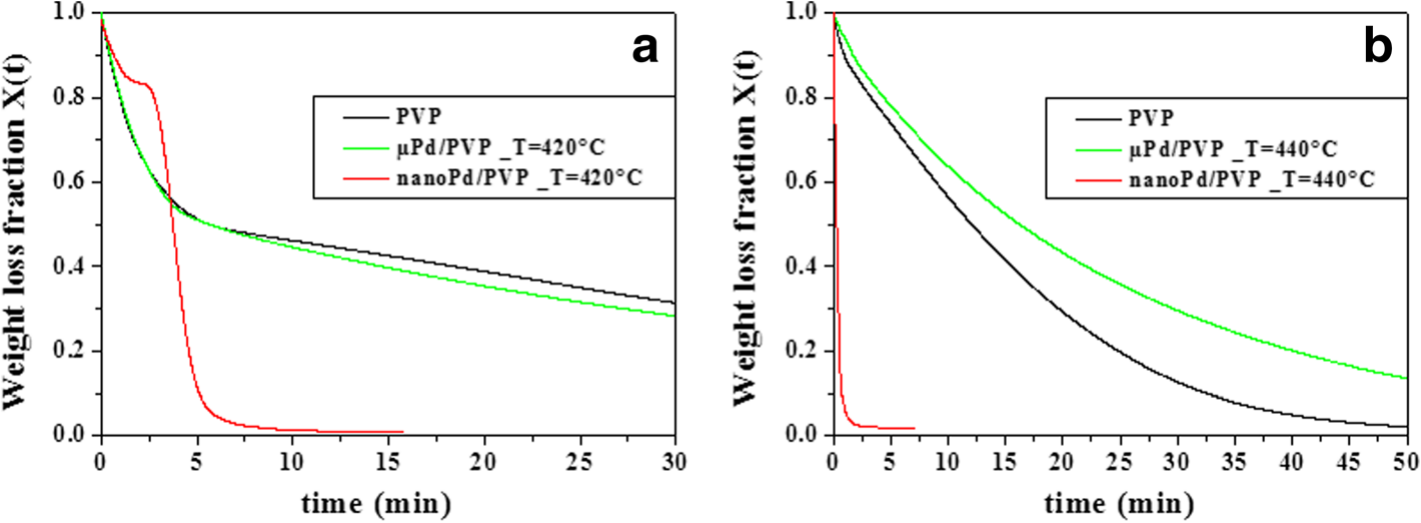
Isothermal weight loss ratio as a function of the time during the combustion of pure PVP (black curves), μ-Pd/PVP (green curves), and nano-Pd/PVP (red curves) at 420 °C (**a**) and 440 °C (**b**)

The nanostructuration of the palladium phase plays an important role in this catalytic combustion process. In fact, the embedding of a micronic Pd(0) powder in the PVP phase, using the same amount of the synthesized nano-Pd/PVP samples (0.3% by weight), does not affect the reaction kinetic (see Fig. 6). In such figure, the weight loss fraction of the pure PVP, of the Pd/PVP sample prepared by using a micronic powder, and of the synthesized nano-Pd/PVP sample are compared. Comparisons are at temperatures of 420 °C (Fig. 6a) and 440 °C (Fig. 6b), respectively. The enhanced catalytic effect of nano-sized Pd compared to the micrometric Pd powder is visually evident. The catalytic activity of the nano-sized Pd increases; thanks to its high specific surface and enhanced catalytic site reactivity.

As a consequence of such a kinetic mechanism (atomic oxygen formation as a rate-limiting step), an effect of the nano-sized Pd catalyst on other polymer combustion could be expected too. Therefore, nano-sized Pd catalyst could be industrially exploited in polymer incineration.

## Conclusions

In this paper, we have investigated the catalytic effect of palladium clusters in the PVP incineration reaction. PVP was selected as the “model polymer” to study the incineration in presence of a catalytic amount (0.3% by weight) of nano-sized Pd(0) clusters (diameter of ca. 2.8 nm). We have found that the polymer incineration performed in presence of nano-sized Pd clusters is promoted by the oxygen atom action, which are more reactive than the molecular form. After a short induction time, it was shown that the presence of nanoscopic Pd(0) catalyst determined an almost instantaneous combustion of the PVP. The isothermal TGA data of the nano-Pd/PVP combustion reaction have been elaborated in order to determine the combustion mechanism, the kinetic constants, the reaction order, and the activation energy. According to the kinetic analysis, a reaction mechanism involving the catalytic formation of atomic oxygen as a zero-order rate-limiting step has been proposed. Nano-sized Pd clusters have shown to exhibit a super-catalytic effect compared to micrometric Pd powder in the PVP incineration. This catalytic effect of nano-sized Pd could be of interest for the incineration of other polymers.

## References

[1] Klabunde KJ (2001). Nanoscale materials in chemistry.

[2] Moser WR (1996) Advanced Catalysts and Nanostructured Materials: Modern Synthetic Methods. Academic Press, San Diego. ISBN 0-12-508460-9.

[3] Doan HA, Sharma MK, Epling WS, Grabow LC (2017). From active-site models to real catalysts: importance of the material gap in the design of Pd catalysts for methane oxidation. Chem Cat Chem.

[4] Du YK, Yang P, Mou ZG, Hua NP, Jiang L (2006). Thermal decomposition behaviors of PVP coated on platinum nanoparticles. J Appl Polym Sci.

[5] Jeong YS, Park JB, Jung HG, Kim J, Luo X, Lu J, Curtiss L, Amine K, Sun YK, Scrosati B, Lee YJ (2015). Study on the catalytic activity of noble metal nanoparticles on reduced graphene oxide for oxygen evolution reactions in lithium–air batteries. Nano Lett.

[6] Stein RS (1992) Polymer recycling: opportunities and limitations 89:835–838. 10.1073/pnas.89.3.835 10.1073/pnas.89.3.835 PMC48336 11607263

[7] Wey MY, Chang CL (1995). Kinetic study of polymer incineration. Polym Degrad Stab.

[8] Park NH, Akamatsu T, Itoh T, Izu N, Shin W (2014). Calorimetric thermoelectric gas sensor for the detection of hydrogen. Methane Mixed Gases Sensors.

[9] Kohl D (1990). The role of noble metals in the chemistry of solid-state gas sensors. Sensors Actuators B Chem.

[10] Eriksson O, Finnveden G (2009) Plastic waste as a fuel—CO_2_—neutral or not? Energy Environ Sci. 10.1039/b908135f

[11] Huang SJ (1995). Polymer waste management—biodegradation, incineration, and recycling. J Macromol Sci Part A.

[12] Lengellé G, Duterque J, Trubert JF (2002). Combustion of solid propellants. Nato.

[13] Chaturvedi S, Dave PN (2013) Solid propellants: AP/HTPB composite propellants. Arab J Chem. 10.1016/j.arabjc.2014.12.033

[14] Zhang M, He F, Zhao D (2015). Catalytic activity of noble metal nanoparticles toward hydrodechlorination: influence of catalyst electronic structure and nature of adsorption. Front Environ Sci Eng.

[15] Rioux RM, Song H, Hoefelmeyer JD, Yang P, Somorjai GA (2005). High-surface-area catalyst design: synthesis, characterization, and reaction studies of platinum nanoparticles in mesoporous SBA-15 silica. J Phys Chem B.

[16] Xian J, Hua Q, Jiang Z, Ma Y, Huang W (2012). Size-dependent interaction of the poly( *N*-vinyl-2-pyrrolidone) capping ligand with Pd nanocrystals. Langmuir.

[17] Bonet F, Delmas V, Grugeon S, Herrera Urbina R, Silvert PY, Tekaia-Elhsissen K (1999). Synthesis of monodisperse Au, Pt, Pd, Ru and Ir nanoparticles in ethylene glycol. Nanostructured Mater.

[18] Teranishi T, Miyake M (1998). Size control of palladium nanoparticles and their crystal structures. Chem Mater.

[19] Evangelisti C, Panziera N, D’Alessio A, Bertinetti L, Botavina M, Vitulli G (2010). New monodispersed palladium nanoparticles stabilized by poly-(*N*-vinyl-2-pyrrolidone): preparation, structural study and catalytic properties. J Catal.

[20] Sugimoto T (2000) Fine particles: synthesis, characterization, and mechanisms of growth. Marcel Dekker

[21] Schiavo L, Carotenuto G, Aversa L, Tatti R, Verucchi R (2016) Synthesis of palladium clusters by reduction of K_2_PdCl_4_ with ethylene glycol. IEEE-NANO 2015 - 15th Int. Conf. Nanotechnol. pp. 282–285. 10.1109/NANO.2015.7388979

[22] Schiavo L, Aversa L, Tatti R, Verucchi R, Carotenuto G (2016) Structural characterizations of palladium clusters prepared by polyol reduction of [PdCl_4_]^2−^ ions. J Anal Methods Chem. 10.1155/2016/9073594 10.1155/2016/9073594 PMC4814673 27073712

[23] Jiménez A, Berenguer V, López J, Sánchez A (1993). Thermal degradation study of poly(vinyl chloride): kinetic analysis of thermogravimetric data. J Appl Polym Sci.

[24] Sait HH, Hussain A, Salema AA, Ani FN (2012). Pyrolysis and combustion kinetics of date palm biomass using thermogravimetric analysis. Bioresour Technol.

[25] Lide DR (2010) CRC Handbook of Chemistry and Physics. 10.1136/oem.53.7.504

